# Spatial protein heterogeneity analysis in frozen tissues to evaluate tumor heterogeneity

**DOI:** 10.1371/journal.pone.0259332

**Published:** 2021-11-19

**Authors:** Anna Fomitcheva-Khartchenko, Maria Anna Rapsomaniki, Bettina Sobottka, Peter Schraml, Govind V. Kaigala

**Affiliations:** 1 IBM Research Europe, Zurich, Rüschlikon, Switzerland; 2 Department of Pathology and Molecular Pathology, University Hospital Zurich and University Zurich, Zurich, Switzerland; Universita degli Studi di Milano-Bicocca, ITALY

## Abstract

A new workflow for protein-based tumor heterogeneity probing in tissues is here presented. Tumor heterogeneity is believed to be key for therapy failure and differences in prognosis in cancer patients. Comprehending tumor heterogeneity, especially at the protein level, is critical for tracking tumor evolution, and showing the presence of different phenotypical variants and their location with respect to tissue architecture. Although a variety of techniques is available for quantifying protein expression, the heterogeneity observed in the tissue is rarely addressed. The proposed method is validated in breast cancer fresh-frozen tissues derived from five patients. Protein expression is quantified on the tissue regions of interest (ROI) with a resolution of up to 100 *μ*m in diameter. High heterogeneity values across the analyzed patients in proteins such as cytokeratin 7, *β*-actin and epidermal growth factor receptor (EGFR) using a Shannon entropy analysis are observed. Additionally, ROIs are clustered according to their expression levels, showing their location in the tissue section, and highlighting that similar phenotypical variants are not always located in neighboring regions. Interestingly, a patient with a phenotype related to increased aggressiveness of the tumor presents a unique protein expression pattern. In summary, a workflow for the localized extraction and protein analysis of regions of interest from frozen tissues, enabling the evaluation of tumor heterogeneity at the protein level is presented.

## Introduction

Intra-tumoral heterogeneity is a common occurrence in breast cancer [1], and has been linked to increased aggressiveness [2, 3] and reduced survival [4]. Changes in the expression of estrogen receptor (ER), progesterone receptor (PR) and human epidermal growth factor receptor 2 (HER2), oncoproteins, epithelial markers and immune system specific proteins highlight the presence of different molecular subtypes within a single tumor [1, 5, 6]. Such heterogeneity hampers an accurate prognosis by biasing the view of the tumor composition based on the analyzed region. This is believed to cause differences in the outcome for breast cancer patients with seemingly similar disease states [7]. The standard way of evaluating protein expression in diagnostics, immunohistochemistry, offers limited duplex possibilities. With a larger number of markers becoming clinically relevant, technologies that allow multiplexing are gaining importance. While immunohistochemistry and immunofluorescence analyses are still the most commonly used techniques in cancer research, they are being displaced by new proteomic methods, including imaging mass cytometry (IMC) [8], imaging mass spectrometry (IMS) [9], or immuno-SABER [10]. Although these techniques offer higher multiplexing capabilities, they require more specialized equipment and, in the case of IMC and IMS, are destructive to the sample, prohibiting follow-up investigation. Alternatively, protein signatures can be obtained using protein microarrays [11] or mass spectrometry [12], although at the cost of losing the tissue structure. To address these challenges, several groups have focused on developing localized tissue extraction strategies, by applying hydrogels containing lysis solutions [13, 14], by cutting out the tissue using laser capture microdissection [12, 15], and by using microfluidic devices [16–18].

The variations observed in protein expression are not bimodal (presence or absence of expression), but often gradient-like [6], highlighting the complexity of the internal state of the cells and their interactions with the microenvironment. Protein abundance at a single-cell level is a continuous variable and establishing a threshold is thus helpful for defining a pathologically high (or low) expression. This strategy is currently employed in most diagnostic laboratories, where semi-quantitative tests take place by visually delimiting the expression of relevant markers by using immunohistochemistry. The reproducibility of this strategy has been challenged [19], with new methods suggesting alternatives for reducing the uncertainty of this type of protein quantification [20, 21]. In research settings, methods such as automated quantitative analysis (AQUA) [22], which are adapted to analyzing fluorescence intensities in a tissue section, offer a more objective metric and means for self-normalization [22]. Other techniques, such as mass spectrometry and microarrays, provide a quantitative metric based on the signal strength relative to other analytes, while an absolute quantification can be obtained through calibration curves [23, 24]. Therefore, having a defined protein quantification strategy is key towards understanding the degree of heterogeneity in a sample. The most commonly employed scores to quantify heterogeneity are the Shannon and Simpson entropy, two biodiversity indices borrowed from ecology that take into consideration both the number of different species and their relative abundance [25–27], but ignore spatial tissue architecture. However, local applications of these metrics can provide insight into spatial tumor heterogeneity, for example by first dividing a tissue into smaller tiles, each with an individual AQUA value, and then assigning a global heterogeneity score to the whole tissue [25]. This method is, however, limited in the number of multiplexed proteins that can be simultaneously achieved due to spectral overlap. Other heterogeneity techniques do take into account the spatial distribution. The H-index, which consists of quantifying the intensity and the proportion of cells expressing this intensity, was developed to evaluate the heterogeneity of HER2 samples [28], albeit using immunohistochemistry. Pointwise mutual information has also been used to study the number and type of cells located in each other’s vicinity [29]. Nevertheless, there is still no consensus regarding the use of a single method or metric, which hinders the comparison of samples across existing studies.

**Fig 1:**
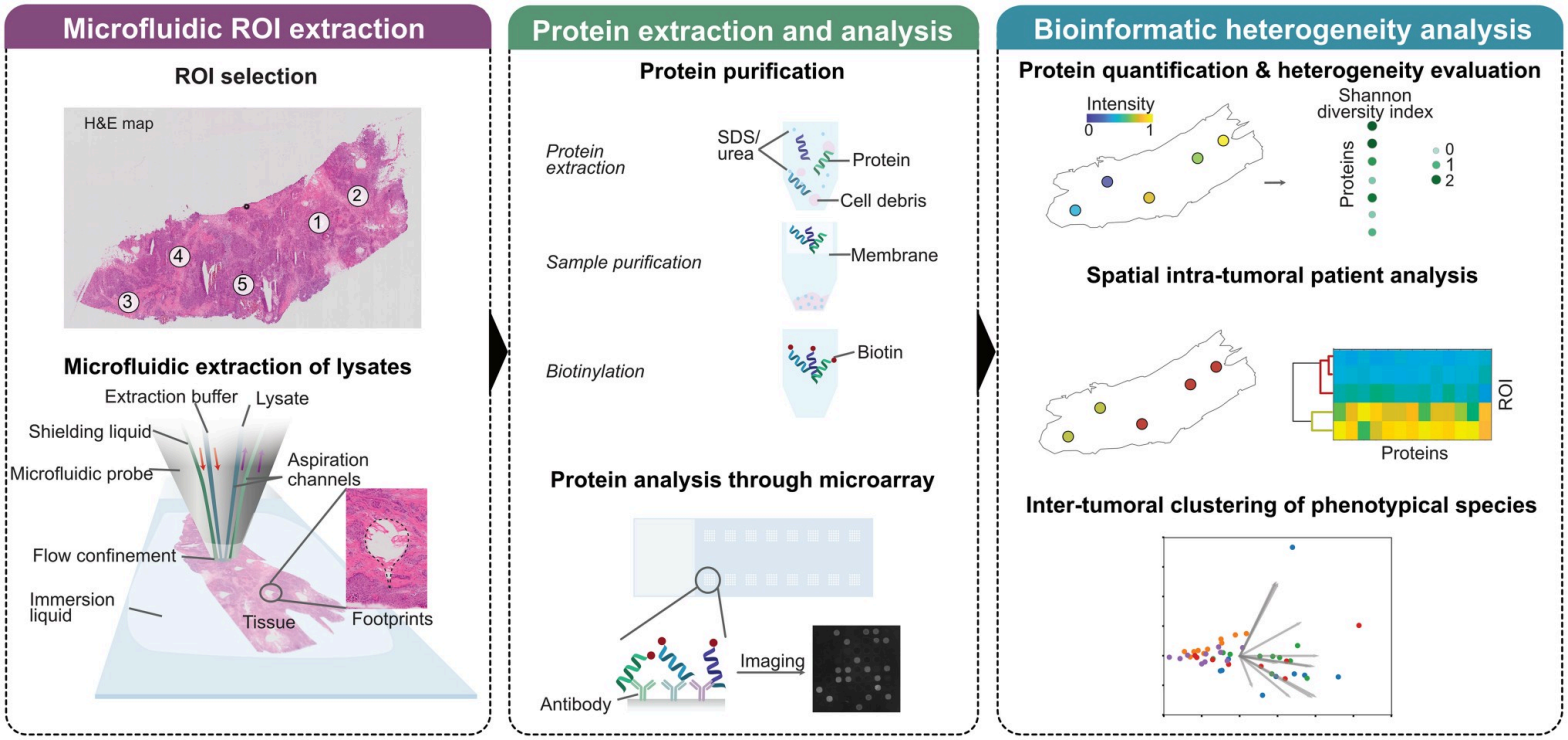
Scheme of the workflow for protein heterogeneity analysis. Using a microfluidic probe, proteins are spatially lysed from a frozen tissue section. The protein is extracted from the lysate, biotinylated and the presence and expression of proteins is analyzed through an antibody microarray. Expression is quantified and the heterogeneity of each protein is evaluated. Finally, inter- and intra- patient heterogeneity is examined.

In this work, we propose a new workflow (Fig 1) to extract proteins from selected regions in frozen tissue samples, analyze the protein content present using antibody microarrays and explore the spatial distribution of phenotypical variants. The extraction of local regions is performed using a microfluidic probe (MFP), a microfluidic device designed to precisely localize chemicals on a few cells [30]. The MFP allows the extraction of small tissue regions of interest, i.e. lysates, and has been previously used to obtain the genetic and transcriptomic footprint of areas of interest in FFPE section [31–33]. We adapted the chemistry of the extraction to lyse frozen tissues and to generate a lysate compatible with a proteomic downstream analysis. The lysate is analyzed using an antibody microarray containing a panel of 13 proteins and the expression of each region is quantified through the intensity of the microarray signal. The final step of this workflow addresses the analysis of spatial heterogeneity for each protein and a clustering of phenotypical variants to unveil spatial heterogeneity both within a patient and across patients in the analyzed population.

## Results

### Workflow for spatial heterogeneity analysis

We created a workflow for spatial extraction of protein and the quantification of heterogeneity at different levels: protein, patient and population. A graphical representation of the workflow is shown in Fig 1. Briefly, regions of interest (ROIs) chosen through a hematoxylin and eosin map from a consecutive tissue section are extracted using an MFP (S1 and S2 Figs). The MFP generates footprints, i.e., extracted ROI areas, with diameters of 100 to 300 μm, each one covering 50 to 100 cells. The size of the footprint can be adapted using different flow rates, while some fluctuations in the size are expected due to local differences in viscosity of the immersion buffer used to avoid tissue drying. Tissue lysis of most of the footprints was achieved within short times (about 20 s), although a longer time was established in this protocol to ensure complete extraction of epithelial cells. Maintaining a constant distance of 20–30 μm between the MFP and the sample is critical to ensure a high extraction success rate, which in our case varied from 80 to 100% among tissues. Once the ROI material is in the lysate (≤20 μL), the protein has to be extracted, purified and biotinylated to undergo protein microarray analysis. To quantify protein abundance, we use the intensity of the corresponding protein spot in the microarray, which we normalize by the area of the footprint to compensate for footprint size fluctuations. We performed the downstream heterogeneity analysis based on these values, as presented in the following section.

### Quantification of protein expression and heterogeneity on spatially distributed regions of interest

**Fig 2:**
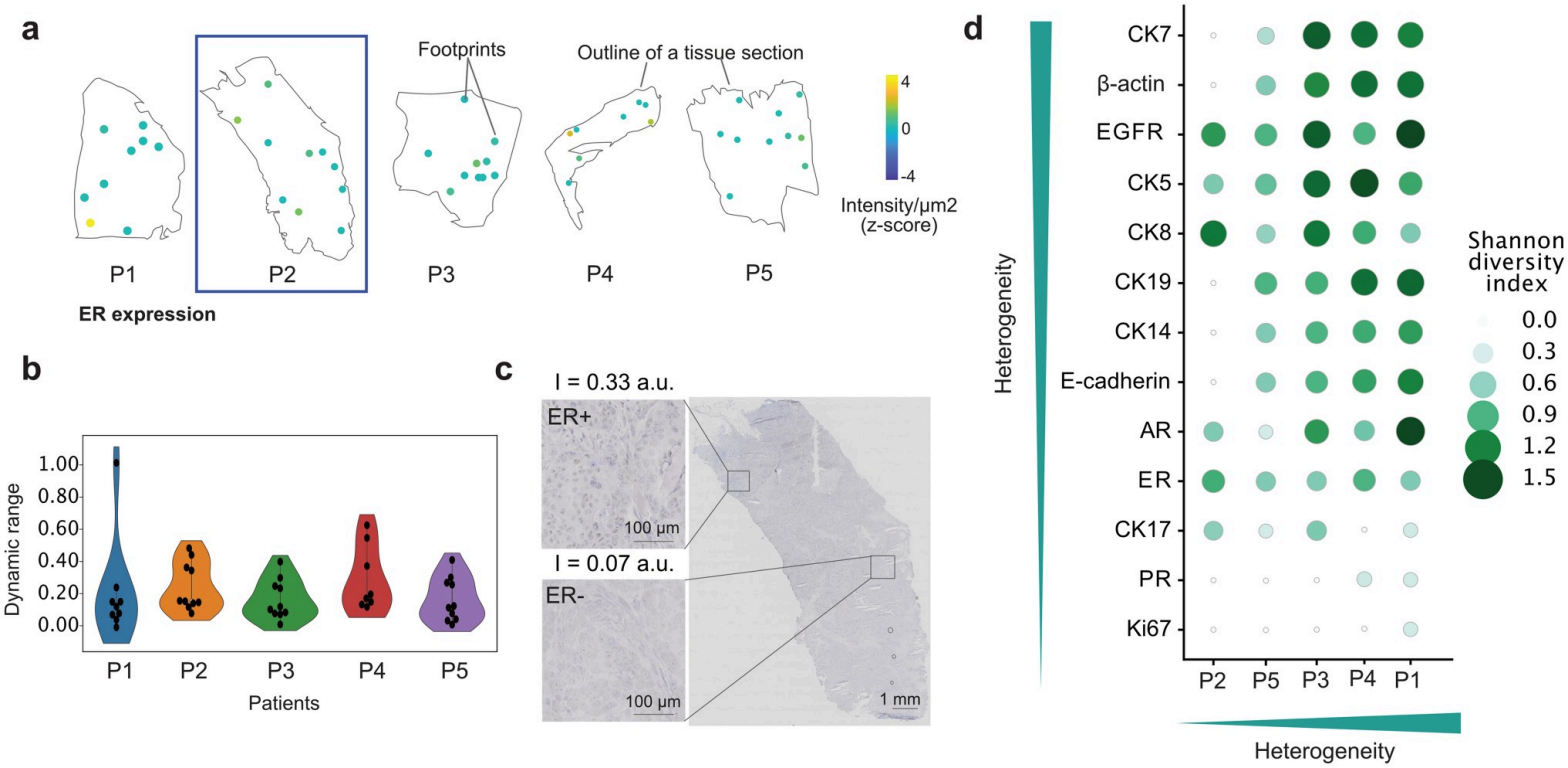
Protein quantification and heterogeneity evaluation. a) The z-score of the intensity for ER is shown for five patients (P1-5). b) Violin plot representing the distribution of the intensities of the proteins across their dynamic range. c) Immunohistochemistry of ER in patient 2. The intensity value (I) corresponds to the footprint located in that same area in a consecutive slide. d) Heterogeneity analysis of all proteins analyzed using the Shannon entropy index. Proteins and patients are sorted by increasing median heterogeneity.

As a first step of the analytical pipeline, we quantified the degree of protein expression as well as the heterogeneity of expression of each protein across the tissue sections. For signal quantification, we define the metric as intensity normalized by unit area (Fig 2a & 2b and S3 Fig), not taking into account the cell count per ROI to avoid bias [34]. The heterogeneity of each protein at a patient level is estimated by using Shannon entropy (Materials and Methods, Fig 2d). In this section, we will briefly outline the results of protein expression and heterogeneity for the housekeeping gene β-actin, steroid receptors, several cytokeratins (CKs), EGFR, E-cadherin and Ki67.

β-actin is a highly conserved protein involved in cell structure, and is often used for normalization, since it is coded by a housekeeping gene. Here, we see variations of β-actin not only across patients but also within the same patient, with patient 1 showing the highest dynamic range of expression. Such results conform with previous reports across different cell lines [35] and within the same patient tissue [33] on the gene and transcript levels, respectively. Interestingly, in our analysis β-actin is one of the proteins that presents the highest heterogeneity score, making it a dubious candidate for protein normalization.

Steroid receptors are a family of nuclear receptors, including PR, ER and androgen receptors (AR). PR and ER are known to exhibit heterogeneous expressions. This potential heterogeneity is already incorporated in the commonly used score for quantifying ER and PR, the Allred score, which analyzes not only the intensity but also the percentage of stained cells in the tumor. ER and PR positive tumors (Allred score 3–8) are typically less aggressive [36, 37], albeit high heterogeneity in ER has been linked to reduced survival in the long term [38], since the presence of ER negative regions could correspond to cells that potentially survive hormonal treatment. All analyzed patients in this work presented a low intensity of PR for the selected ROIs, with patients 1 and 4 showing one single ROI with ‘positive’ signal. In the case of ER, the overall heterogeneity was low, with all patients presenting a similar dynamic range of expression. The extent of the variations observed using our method was comparable to those in immunohistochemistry (Fig 2c and S3 Fig). The third analyzed steroid receptor, AR, tends to be expressed in the same tissues as ER [36], showing anti-proliferative effects in ER+ tumors [39]. Here, AR presented higher heterogeneity in patients 1 and 3. A correlation between AR and ER expression was also observed (S4 Fig).

CKs are proteins composing the cytoskeletal intermediate filaments of epithelial cells. While there is a large number of CKs present in cells, we selected CK19, CK17, CK14, CK8, CK7 and CK5 due to their involvement in breast cancer. CK19 presented the highest dynamic range for the examined patients. CK19 is a luminal epithelium cytokeratin, often present in healthy breast tissue and expressed in ~90% of breast cancers. Its absence has been linked to increased aggressiveness in breast cancer [40], as it might have a role in attenuating cancer hallmarks such as migration or proliferation [41]. In this study, all patients were positive for CK19, albeit with different degrees, with the exception of patient 2, who had both a low expression and low heterogeneity of CK19. Basal cytokeratins CK17 and CK5 are known indicators for poor prognosis [42], quick disease progression and lower overall survival [43, 44]. Our results indicate low heterogeneity for CK17, while CK5 was among the proteins with the highest degree of heterogeneity, especially for patients 3 and 4. The last examined basal cytokeratin, CK14, is commonly present in the basal layer of healthy tissue [44, 45], as shown in the CK14 negative inset of S3 Fig. CK14 has been associated with increased survival rates when no metastasis is present, although in patients with metastatic cancer the opposite trend is observed [46]. In our analysis, we see a high dynamic range in patients 1, 3 and 4. The expression in patient 1 is heterogeneous, as confirmed by immunohistochemistry, where focal staining is observed (Fig 2c). CK8 is a luminal cytokeratin linked to a resistance to chemotherapy and related to the resistance to tumor necrosis factor-related apoptosis-inducing ligand (TRAIL) induced apoptosis, product of its interaction with the death receptor 5 [47]. Here, CK8 is heterogeneously expressed, especially for patients 2, 3 and 4. The role of CK7 in breast cancer has been less investigated, although the pattern CK7/CK20 seems to be widely expressed in triple negative cancers, with CK7 staining often being heterogeneous [48]. In this study we also observed high heterogeneity in CK7, it being the overall most heterogeneous protein observed in the analyzed sample, with large variations of expression in patients 1, 3 and 4.

EGFR, a transmembrane protein, often serves as a marker associated with larger tumor sizes, poor differentiation and poor prognosis in breast cancer [49]. In our cohort, patient 1 exhibits the largest dynamic range of expression of this protein, with a high heterogeneity in patients 1 and 3. Contrary to EGFR, an abnormal expression of E-cadherin implies a reduction in its intensity, as its complete or partial loss correlates with tumor invasion and metastasis [49]. Nonetheless, the metastatic site tends to show normal E-cadherin expression levels, independently of its expression in the primary tumor [50]. In our cohort this could be the case for patient 2, who presented the lowest expression uniformly across the tissue. However, from the pathology report we know that it did not migrate to the lymph nodes at the time of analysis.

The last protein analyzed, Ki67, is a nuclear protein involved in cell proliferation and used to establish patient prognosis. Ki67 expression is often heterogeneous, with tumors that show over 20% of expression being correlated with worse prognosis [51]. We found low expression of Ki67 in all patients except for one ROI in patient 1. Such a pattern is not surprising, given the discretization of the ROI extraction in this study.

Overall, patient 1 presented the highest global heterogeneity (Fig 2d), while patient 2 was the most homogeneous. On the protein level, we observed that CK7, β-actin and EGFR showed the highest heterogeneous expression patterns across all the patients, while Ki67 and PR showing the lowest, due to the overall low expression in the analyzed tissue sections.

### Spatial dispersion of phenotypical variants across patients

**Fig 3:**
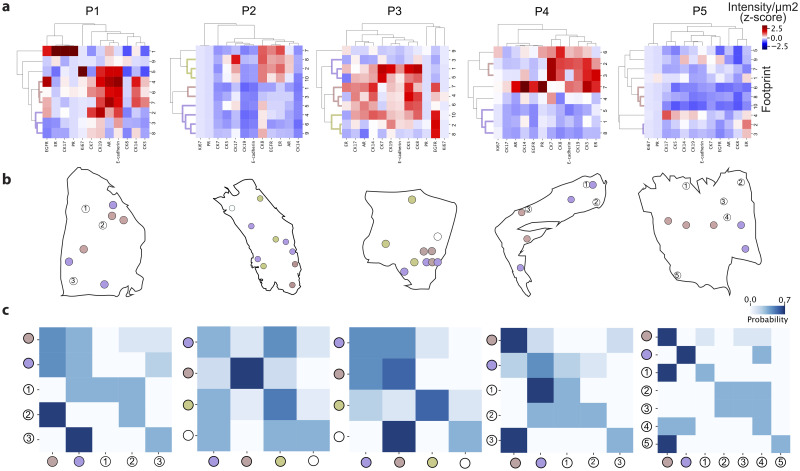
Intra-tumoral heterogeneity analysis. a) Protein expression clustering to evaluate inter-tumor heterogeneity. b) Spatial location of the clusters. The coloring of the clusters in each patient is independent from the other patients. c) Spatial heterogeneity analysis. The heatmap indicates spatial cluster co-occurrence in the 3 nearest ROIs, as described in Materials and Methods.

Several breast cancer molecular ecosystems can co-exist within the same patient [6]. We have thus decided to explore the molecular ecosystems that exist within a single patient. Using hierarchical clustering on the intensity levels of all proteins (β-actin was not included in the analysis), we found that each patient had 2 to 3 distinct clusters with common phenotypical expression (in brief, phenotypical clusters), with the rest of the footprints forming their own singleton variant (Fig 3a). While the clusters of patients 1, 3 and 4 included higher levels of expression for several proteins, patients 2 and 5 were mostly characterized by low protein expression. This is also the case for one of the clusters in patient 4 (purple cluster).

Since our workflow allows us to preserve the spatial distribution of the tissue and each phenotypical cluster could now be spatially located, we further assessed whether areas of similar molecular profiles were also spatially clustered. A visual assessment (Fig 3b and S5 Fig) indicated that, although for some patients the footprints belonging to the same cluster appeared to be spatially colocalized (e.g., red cluster in patient 3), in multiple cases the pattern was more dispersed. Indeed, we computed the Spearman correlation coefficient between physical proximity (Euclidean distance between footprint coordinates) and proximity in the molecular space (Euclidean distance between molecular intensity) for all patients, resulting in correlation values of 0.13, 0.23, 0.06, -0.01 and 0.19 for patients 1 to 5, respectively, which are all statistically not significant (p-values = 0.45, 0.14, 0.68, 0.94 and 0.20 for patients 1 to 5, respectively).

To assess spatial heterogeneity, we computed heatmaps of cluster spatial co-occurrence using a *k*-nearest neighbor approach (Materials and Methods, Fig 3c). We can see that in some cases, such as patients 2 and 5, some variants are spatially close, possibly indicating a common evolutionary origin. Computing a global spatial heterogeneity metric indicated that patient 3 was the most spatially homogeneous and patient 1 the most spatially heterogeneous (scores 0.36, 0.46, 0.47, 0.42 and 0.43 for patients 1 to 5, respectively). In the cases of patients 1 and 3 the analyzed regions correspond to in situ ductal carcinoma (DCIS, S6 Fig). Since the tissue sections are a representation on a single plane from a 3D tissue, it is possible that the observed ducts are connected, even if the variants seem further apart in the analyzed slide. This is especially visible in patient 3, where several close-by ducts present the same expression pattern. Patients 2 and 4 present a high grade (G3) invasive ductal carcinoma. We believe this to be the cause of the large spatial distance between the phenotypical clusters, either because the phenotypically close cells are pushed apart by newly forming ones, or because of a fast mutation rate. Nevertheless, a localized genetic analysis would be required to confirm any of these hypotheses. Patient 5 also presents an invasive ductal carcinoma, although in this case the phenotypical clusters are in visual proximity, albeit not statistically significant.

### Intra-tumoral heterogeneity analysis for clustering across patients

**Fig 4:**
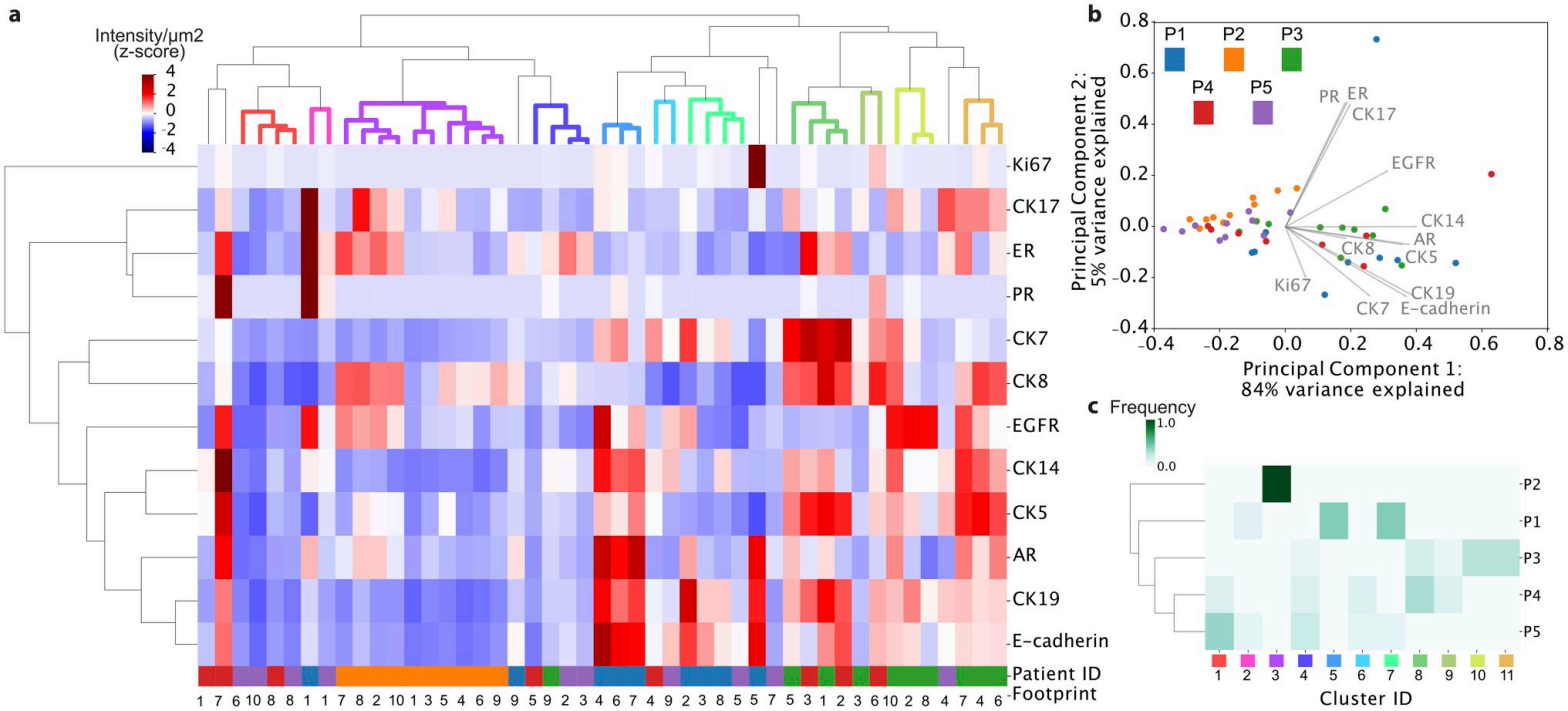
Inter-tumoral heterogeneity analysis. a) Hierarchical clustering showing the phenotypical variants across patients and protein correlations. b) Principal component analysis (PCA). c) Similarity of clusters between patients.

The final step in the proposed workflow was the evaluation of the heterogeneity present across the analyzed population. Across the existing phenotypical clusters, we expect some of them to be common across different patients. Four of the analyzed patients indeed had common clusters. However, when compared to the others, patient 2 presented a unique phenotype (Fig 4a and 4c), clustering in one single group with low heterogeneity and low protein intensity. The uniqueness of patient 2 could be indicative of a more aggressive phenotype [6], further indicated by the low expressions of CK19 and E-cadherin, also related to higher tumor aggressiveness [40, 49]. This cluster contains three subgroups, one with a luminal phenotype described by a high expression of ER and CK8, while the second one lacks the expression of ER, and the last, also that of CK8. From our cohort, patients 4 and 5 are the most phenotypically similar patients, sharing clusters 1, 4 and 6, mostly characterized by low protein expression. Patient 4 also shares clusters 8 and 9 with patient 3. These clusters show a high expression of CK7, 8, 5, 19 and E-cadherin. Nevertheless, patient 3 presents two subpopulations with a basal phenotype (CK14 and 5 overexpression, clusters 10 and 11). Patient 1’s sample is dominated by clusters 5 and 7, with high expressions of EGFR, CK14, CK5, AR, CK19 and E-cadherin, while sharing cluster 2 with patient 5.

When looking at the principal component analysis (PCA, Fig 4b), a similar tendency is observed. Patients 2 and 5 have their ROIs clustered together in a region of lower expression, while some footprints of patients 1, 3 and 4 show strong correlations, corresponding to the heterogeneity observations of the Shannon entropy analysis.

## Discussion

The nature of tumor heterogeneity requires precise tools to understand its extent and biological relevance. The spatial analysis of tumors can give new insights into the generation, proliferation and evolution of tumors with respect to the normal tissue architecture, critical towards understanding disease progression and improving patient prognosis. In this work, we present a novel workflow that performs a quantitative analysis of protein expression and highlights the extent of heterogeneity within a tumor section. Using an open-space microfluidic system, we achieve a quick lysate extraction compatible with commonly used microscopy slides. We envision this system to be totally automatable, thus reducing the total extraction time and increasing the number of analyzed ROIs per section. While here we focus on the extraction of ROIs of small diameter, it is in principle possible to reduce the confinement to cover a single cell by changing the design of the channels of the MFP [52], allowing additional types of investigations, such as the analysis of intra-cellular protein concentration [53, 54]. For the downstream processing, we use a well-established technology to perform the analysis—antibody microarrays. Microarrays underwent a boom in the early 2000s due to their high degree of multiplexing and sensitivity. Currently, their application has been rather reduced, likely due to the advent of mass spectrometry, although their simplicity of use and high dynamic range make them ideal for examining focused protein panels. Additionally, we incorporate into this workflow strategies to quantify heterogeneity by evaluating spatially distributed protein expression to explore the extent of phenotypical changes happening in the tumor.

In the analytical front, the proposed method allows the exploration of different clusters that can be related to cancer subtypes across micrometer- and millimeter-sized regions. These clusters showed a disperse distribution even in spatially close regions, which could be explained by the spatial distribution of milk ducts on a 2D surface (patients 1 and 3), and by varying microenvironments or rapidly multiplying clones in the rest of the patients (patients 2 and 4). In the literature, cancer subtypes have been explored for both genomic and proteomic data, although the two types of subgroups do not necessarily cluster the same way [55]. The causes for that include local gene expression variations due to several factors such as the microenvironment, and not all the mRNA translating into protein [56]. In our work, we identified 11 proteomic clusters, most of them being common across the patients, with the exception of a unique cluster present in patient 2, which presented characteristics linked to increased aggressivity.

For this workflow we used fresh-frozen sections. While this type of tissue sections is still rare due to storage price, it offers several advantages over formalin-fixed paraffin-embedded (FFPE) sections. Fixing FFPE sections often involves non-standardized protocols, which can have vast differences in processing in tissues of different sizes [57]. Furthermore, the mechanism of antigen retrieval, necessary to remove the chemical modifications added by formalin, is not well-understood, with large variations in buffer composition and time incurring for each protein [58]. This introduces an important degree of uncertainty about the amount and quality left of the protein of interest. Conversely, frozen sections present proteins in their native state, with biological activity still present in some cases [59]. This tissue conservation method is also preferred for DNA and RNA analysis, since less fragmentation of these nucleic acids is expected [60]. Thus, we envision this work as a step towards proteomic integration with genomic analysis, which was already demonstrated on the microscale [32]. Steps towards the integration of several ‘omic’ modalities on tissue sections have been undertaken [61], albeit using larger sample regions over greater distances, thus overlooking microscale intra-tumoral heterogeneity.

The exploration of protein heterogeneity offered by our workflow demonstrates the relevance and necessity of spatially localized methods to explore the proteomic landscape. The analytical workflow we designed makes data easily accessible, facilitating the visualization of the different phenotypical variants in a patient. Thus, we envision this method to have a role in easy and multiplexed protein heterogeneity data evaluation.

## Methods

### Tissue sections

Five patient samples were provided by the Department of Pathology and Molecular Pathology, University Hospital of Zurich. The tissues were samples of primary invasive ductal carcinoma (IDC) of the breast including ductal carcinoma in situ (DCIS) as the precursor lesion (see S1 Table for pathological details and analyzed regions). All sections were cut with a thickness of 10 μm and deposited onto SuperFrost plus slides.

### Ethics statement

Our retrospective study fulfilled the legal conditions according to Article 34 of the Swiss Law “Humanforschungsgesetz” (HFG), which allows the use of biomaterial and patient data for research purposes without informed consent, if i) it is impossible or disproportionately difficult to obtain patient consent; ii) there is no documented refusal; iii) research interests prevail the individual interest of a patient. Law abidance of this study was reviewed and approved by the ethics commission of the Canton Zurich (BASEC-No. 2019–01477).

### Microfluidic probe fabrication and microfluidic platform set-up

The MFP was fabricated as previously described [30]. Briefly, a mask with the channel pattern was written using a mask writer. Then, channels with a depth of 50 μm were etched onto a silicon wafer using deep reactive ion etching. The silicon wafer was anodically bonded to a glass wafer and the resulting wafer was diced to obtain individual MFP heads. The apex of the head was polished to remove any imperfections that could disturb the flow. Prior to use, the head channels were flushed successively with isopropanol, ethanol and water. The MFP head was then connected to glass syringes (Hamilton, Bonaduz, Switzerland) through corresponding connectors. The MFP head was mounted on motorized stages (LANG, Reiden, Switzerland) located on top of an inverted microscope (Nikon, Egg, Switzerland) (S1 Fig).

### Extraction of region of interest from tissues

The tissues were thawed from -80°C to room temperature and then immersed in an OCT:PBS 8:3 solution (OCT compound from VWR, Dietikon, Swtzerland; PBS from Gibco, ThermoFisher Scientific, Reinach, Switzerland) to avoid detachment from the slide during processing. Regions of interest were probed with an MFP. Owing to the local changes in viscosity of the immersion medium, flow rates were adjusted locally, with the following flow rates being the default values: 2:5:-5:-8 μL/min for shielding injection: processing injection: tissue aspiration: shielding aspiration. Aspiration was kept constant to allow for complete collection of the tissue lysate. The shielding solution contained PBS with red colorant for the visualization of the confinement. The lysis solution consisted of 2% sodium dodecacylsulfate (SDS, Sigma-Aldrich, Buchs, Switzerland) and 8M urea (Sigma-Aldrich, Buchs, Switzerland) in PBS. The lysis time was set to 3 min to ensure the complete dissolution of the tissue. After lysis, the liquid containing the tissue was collected from the corresponding aperture. This lysate was then diluted with lysis solution up to a volume of 50 μL and left to incubate for protein extraction for at least 30 min at 4°C.

The tissue was cleaned of the immersion buffer and submerged in acetone at -20°C for 15 min. The slide was then air-dried for 10 min and submerged in PBS for rehydration. Subsequently, it was transferred to hematoxylin and stained during 5 min. The excess hematoxylin was washed off using running tap water for 3 min and the slide was deposited in HCl 1% for 10-20s to reduce hematoxylin overstaining. After another washing with water, slides were submerged in eosin for 1 min. The slide was then dehydrated in ethanol and mounted.

### Protein preparation

The lysate was centrifuged for 15 min at 14,000 xg and 4°C. The supernatant was diluted with 200 μL of Tris-HCl 20 mM (Sigma-Aldrich, Buchs, Switzerland) and incorporated into an amicon ultra centrifugation column with a pore size of 10 kDa (Merck, Zug, Switzerland). The lysate was centrifuged for 10 min at 14,000 xg and room temperature. A buffer exchange to PBS was then performed by adding 200 μL of PBS to the purification column. This buffer exchange step is critical to remove excess SDS, which has been shown to reduce antigen to antibody binding [62]. A centrifugation was performed until the final volume inside the column reached 20 μL. The lysate was biotinylated using a biotynilation kit (abcam, Cambridge, UK) and following the instructions given by the provider.

### Microarray

A custom microarray with a panel containing 13 proteins was purchased (Sciomics, Neckargemund, Germany). Proteins used in the microarray were: β-actin, cytokeratin (CK) 19, CK14, CK17, EGFR, E-cadherin, ER, PR, Ki67, AR (R&D Systems, Abigton, UK), CK8/18 and CK7 (abcam, Cambridge, UK), and CK5/6 (Merck, Zug, Switzerland), all spotted at a final concentration of 0.5 mg/mL. The microarray was incubated with 10% non-fat dry milk in PBS for 30 min and washed with 0.05% Tween-20 in PBS (PBST) three times for 5 min. The biotinylated protein was then diluted in 1% bovine serum albumin (BSA, Sigma-Aldrich, Buchs, Switzerland) in PBS to 1/10^th^ of the initial concentration. 50 μL were incubated in the microarray for 1 h and then washed with PBST three times for 5 min. A solution of Alexa555-streptavidin (Life Technologies, Bleiswijk, Netherlands) at a concentration of 1:500 in 1% BSA in PBS was incubated for 30 min and washed twice with PBST and once with distilled water. The microarray was then dried through centrifugation and stored in the dark until imaging.

### Immunohistochemistry

Frozen tissues were fixed in acetone at -20°C for 15 min. The slide was then air-dried for 10 min and submerged in PBS for rehydration. A peroxidase blocking agent (abcam, Cambridge, UK) was added for 5 min and washed with a wash buffer (Dako, Basel, Switzerland). The slide was blocked with 1% BSA in PBS for 30 min and washed with wash buffer. Primary antibodies were incubated for 1 hour (anti-CK14 and anti-ER at 15 μg/mL, both from R&D Systems, Minneapolis, US). After a wash, a secondary antibody was added for 30 min (visualization reagent, Dako, Basel, Switzerland) and washed with the wash buffer. Finally, a freshly prepared 2–2’-diaminobenzidine (DAB) solution was left on the sample for 10 min for color signal development and washed away with water. The slides were counterstained with hematoxylin as described in Footprint extraction.

### Imaging

Slides were imaged via a slide scanner (Sciomics, Neckargemund, Germany). The resolution of the imaging was 5 μm, with a gain of 50000 in the green channel and a laser power of 50%. Saturated spots were imaged using a gain of 1000.

### Data analysis

The grey intensity of the microarray spots was measured using custom scripts in MATLAB (S7 Fig). The area of each footprint was calculated based on the image using the Fiji area measurement tool (S8 Fig). The statistical analysis of the data was performed in Python using the numpy, scipy, pandas, scikit-learn and seaborn software libraries.

### Heterogeneity quantification

For the heterogeneity analysis (Fig 2d), we first performed a min-max normalization to scale the raw intensity of each protein to [0,1] as follows:

$$x_{i,j}^{norm}=\frac{x_{i,j}-min(x_{j})}{\text{max}\left(x_{j}\right)-min(x_{j})}$$

where *x*_*i*,*j*_ is the intensity of protein *j* in footprint *i*. We then discretized each vector of protein intensities *x*_*j*_, using *K* = 5 equal-width bins, and computed the Shannon entropy *H* for each protein *j* and each patient *p* as follows:

$$H_{j,p}=-\sum_{k=1}^{K}p_{j,p,k}\text{log}p_{j,p,k}$$

where *p*_*j*,*p*,*k*_ is the relative abundance of protein *j* in patient *p* in bin *k*.

### Intra-patient clustering

Raw protein intensities were initially normalized using a z-score normalization as follows:

$$x_{i,j}^{z-score}=\frac{x_{i,j}-\overset{-}{x_{j}}}{s_{j}}$$

where $\overset{-}{x_{j}}$ and *s*_*j*_ denote the sample mean and standard deviation of each protein respectively, computed across all footprints. Data was clustered using hierarchical clustering (Fig 3a), with average linkage and the Pearson correlation as a distance metric. Footprints were assigned to different clusters using a distance threshold criterion (Fig 3b).

### Spatial heterogeneity analysis

To compute the heatmaps of spatial heterogeneity for each patient (Fig 3c), we followed an approach based on *k*-nearest neighbors (*k*-NN), as previously described [6]. Briefly, for each patient, we have *i* = 1, …, *N* footprints, each one assigned to *c* = 1, …, *C* clusters. We first construct a *k*-NN graph where each footprint is connected to the *k* = 3 most proximal footprints, computed using the Euclidean distance between footprint coordinates. For each footprint *i* we retrieved the cluster labels of the 3 nearest neighbors and assessed the neighbors’ vote, *i*.*e*., the frequency of all *C* clusters in the 3 neighbors. This yielded an *N* × *C* matrix, where each value represents the probability that footprint *i* belongs to cluster *c*. For footprints assigned to the same cluster, we computed the mean of all corresponding rows, resulting in a *c* × *c* matrix that expresses similarities between footprints based on their physical proximity in the tissue. Values on the diagonal of this matrix represent how “self-contained” each cluster is in the tissue, and off-diagonal values represent how much this cluster is intermixed with other clusters. A spatial heterogeneity score for the whole tissue is then simply obtained by dividing the sum of all diagonal elements by the sum of all matrix elements.

### Inter-patient clustering

Z-scaled data across all patients were first clustered using hierarchical clustering, with average linkage and the Pearson correlation as a distance metric (Fig 4a). For each patient, we estimated the percentage of this patient’s footprints belonging to each identified cluster, resulting in a probability distribution across all clusters that sums up to 1. These frequencies were in turn clustered using the Jensen Shannon Divergence (JSD) metric, an appropriate distance metric to measure the similarity between probability distributions that is symmetric and bounded between 0 and 1. Let *P*, *Q* denote the probability density of patients *p*, *q* over all clusters. The JSD between patients *p*, *q* is defined as follows:

$$JSD(P|\left|Q\right)=\frac{1}{2}D_{KL}(P|\left|M\right)+\frac{1}{2}D_{KL}(Q||M)$$

where $M=\frac{1}{2}\;\left(P+Q\right)$ and *D*_*KL*_ is the Kullback-Leibler (KL) divergence:

$$D_{KL}(P|\left|Q\right)=\sum_{i}P_{i}log\frac{P_{i}\;}{Q_{i}}.$$

### Batch effect evaluation

We evaluated the data to explore the presence of potential batch effects in the data as described in [63]. No potential batch effects were identified.

## Supporting information

S1 Fig Microfluidic probe set up. Top left represents an MFP head and bottom left shows the functionality of the head. The right site of the figure shows an MFP mounted on top of an inverted microscope and connected to syringes.
(TIF)

S2 Fig Extraction of a footprint. a) Panel showing the process of a footprint extraction, comprising area selection (t = 0 min), confinement generation (t = 1 min), and visual evaluation of the footprint (t = 3 min). b) Example of a footprint, where epithelial cells have been removed, while matrix is still in place.
(TIF)

S3 Fig Details of analysis for all analyzed proteins. The z-score of the intensity for the analyzed footprints for five patients (P1-5) next to a violin plot representing the distribution of the intensities of the proteins across their dynamic range. An IHC analysis of CK14 is shown for patient 1.
(TIF)

S4 Fig Correlations between proteins. (TIF)

S5 Fig Spatial heterogeneity on tissues. Line thickness is inversely proportional to molecular distance.
(TIF)

S6 Fig Image of the analyzed tissues with a close up on the right of the footprints used for the analysis. The dotted line represents the area where the footprints are located. Scale bar: 1 mm on the tissue (left) and 200 μm for the footprint close ups (right). The numbers represent the numeration of the footprints and their location on the tissue is shown with a grey scale.
(TIF)

S7 Fig Algorithm for array analysis and quantification. A MATLAB-based algorithm was developed to perform the analysis of the gray scale intensity of the microarray. The original image of the array was uploaded and cut into subarrays. The location of two reference spots was manually adjusted (Fig panel A) and a template of a 9x9 array was placed on top of the array (panel B) and visually confirmed to correspond to the location of the spots. A mask was located on each spot of the array (panel C) and using Otsu thresholding the spot was adjusted (panel D). The gray intensity of each spot was then calculated. Spots presenting artifacts were removed from the analysis.
(TIF)

S8 Fig Analysis of the area of the footprints. a) Definition of area of the footprint, the X and the Y-axis for analysis. Extracellular matrix was excluded from the area considerations. b, c and d) Boxplot representing the area, X-axis and Y-axis of the footprints for each patient, respectively. Red crosses represent the outliers.
(TIF)

S1 Table Disease staging and histopathology of the patients. (PDF)

S2 Table Grey intensity measurements for the five patients. (XLSX)
