# Supplementary figures and images for: Spatial protein heterogeneity analysis in frozen tissues to evaluate tumor heterogeneity
Source: PLoS One. 2021 Nov 19;16(11):e0259332. doi: 10.1371/journal.pone.0259332 (PMC8604290; doi:10.1371/journal.pone.0259332)

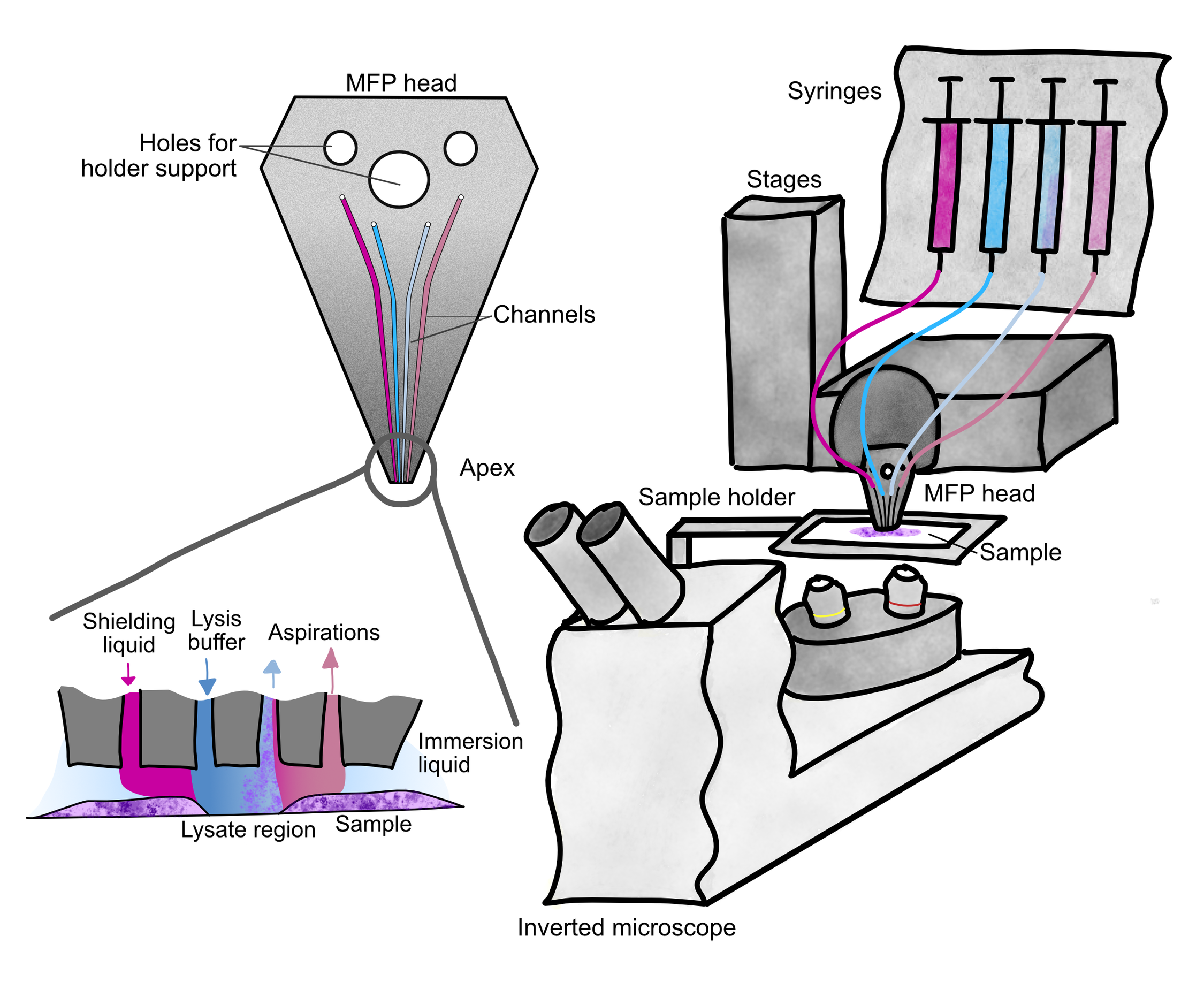

Supplement: S1 Fig — Top left represents an MFP head and bottom left shows the functionality of the head. The right site of the figure shows an MFP mounted on top of an inverted microscope and connected to syringes. (TIF) [file pone.0259332.s001.tif]

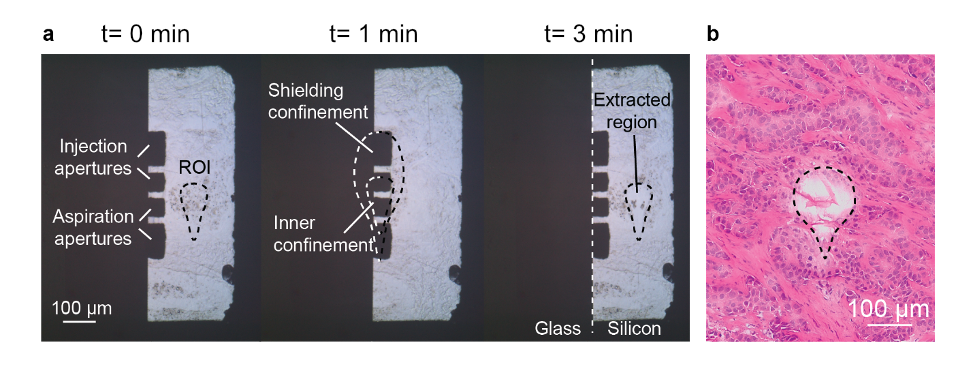

Supplement: S2 Fig — a) Panel showing the process of a footprint extraction, comprising area selection (t = 0 min), confinement generation (t = 1 min), and visual evaluation of the footprint (t = 3 min). b) Example of a footprint, where epithelial cells have been removed, while matrix is still in place. (TIF) [file pone.0259332.s002.tif]

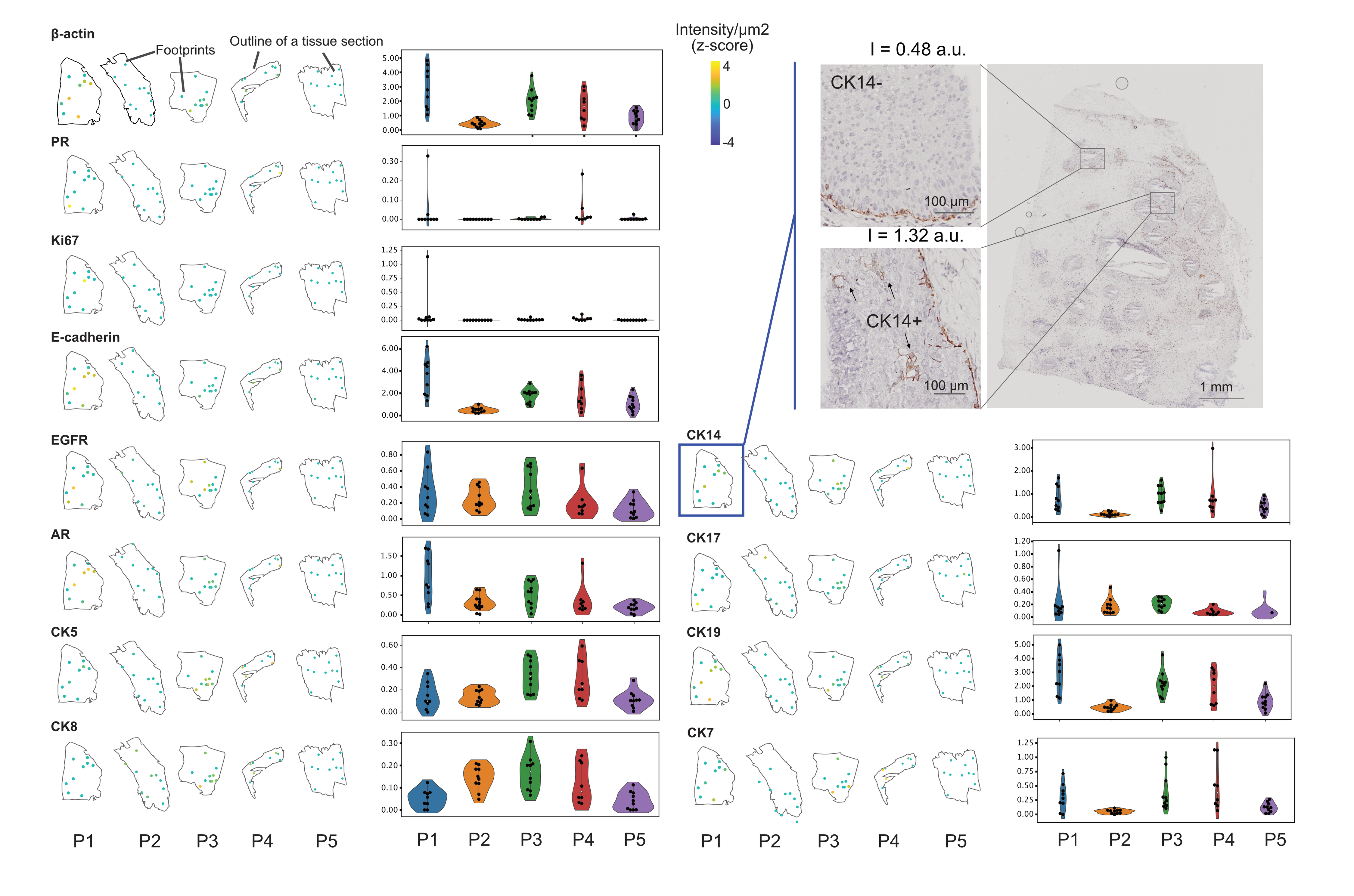

Supplement: S3 Fig — The z-score of the intensity for the analyzed footprints for five patients (P1-5) next to a violin plot representing the distribution of the intensities of the proteins across their dynamic range. An IHC analysis of CK14 is shown for patient 1. (TIF) [file pone.0259332.s003.tif]

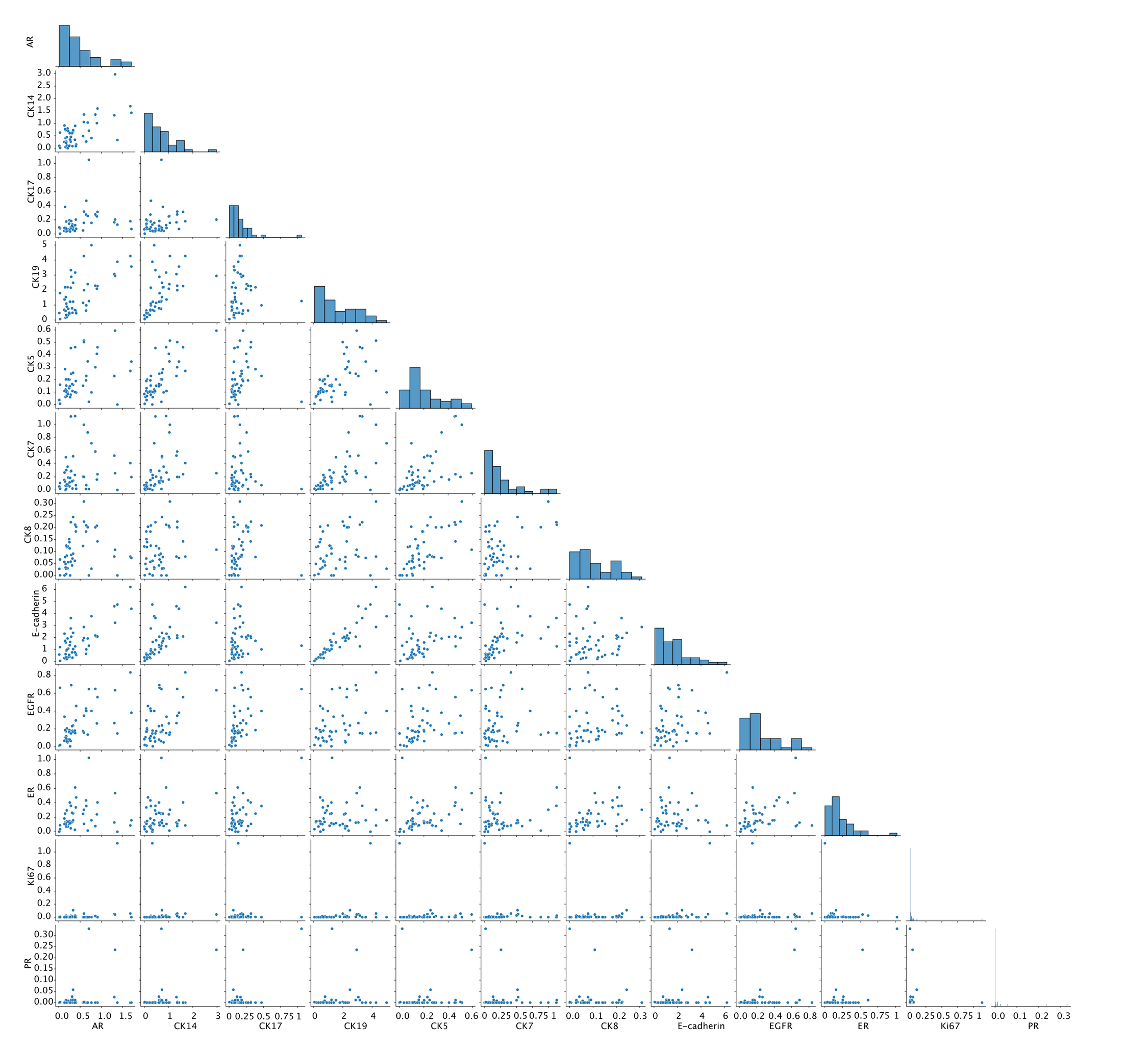

Supplement: S4 Fig — (TIF) [file pone.0259332.s004.tif]

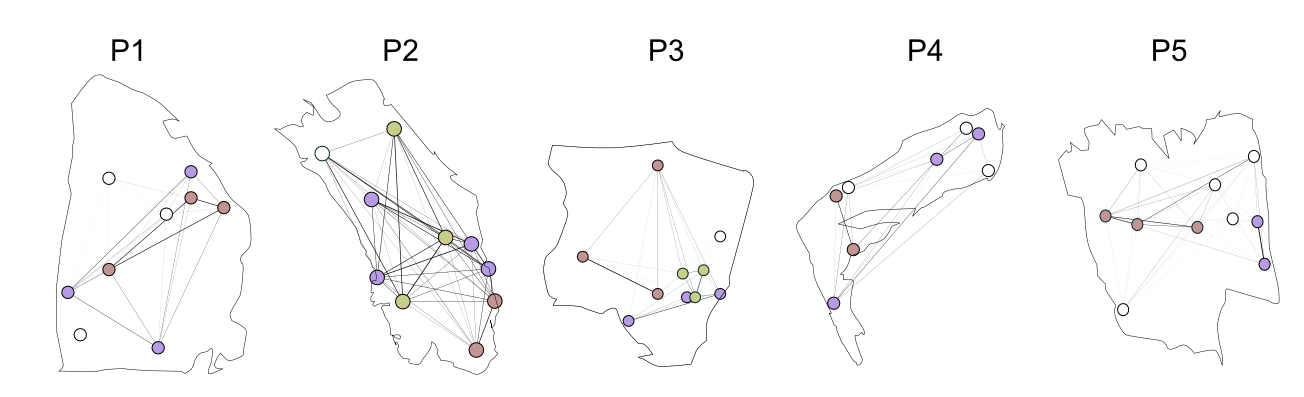

Supplement: S5 Fig — Line thickness is inversely proportional to molecular distance. (TIF) [file pone.0259332.s005.tif]

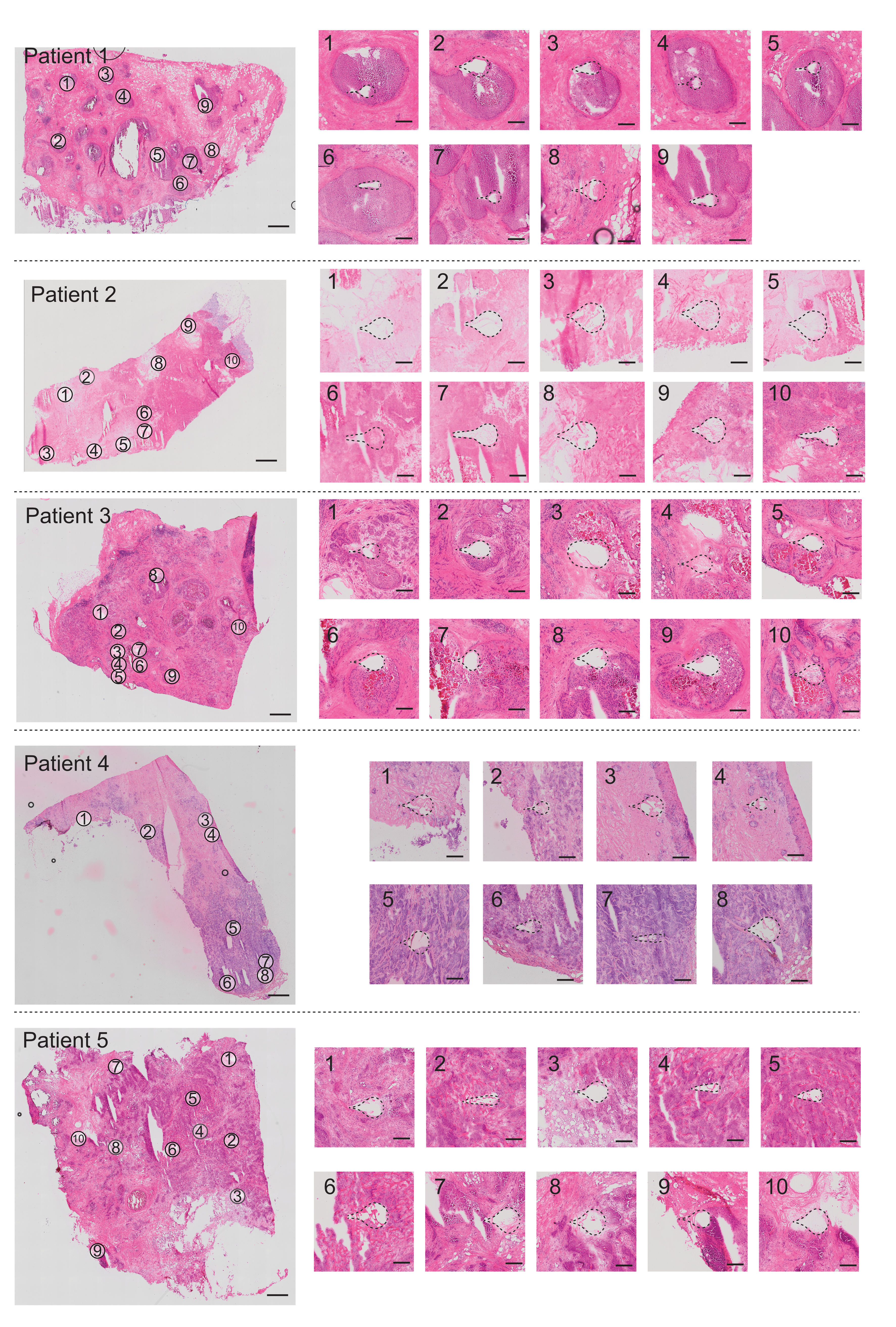

Supplement: S6 Fig — The dotted line represents the area where the footprints are located. Scale bar: 1 mm on the tissue (left) and 200 μm for the footprint close ups (right). The numbers represent the numeration of the footprints and their location on the tissue is shown with a grey scale. (TIF) [file pone.0259332.s006.tif]

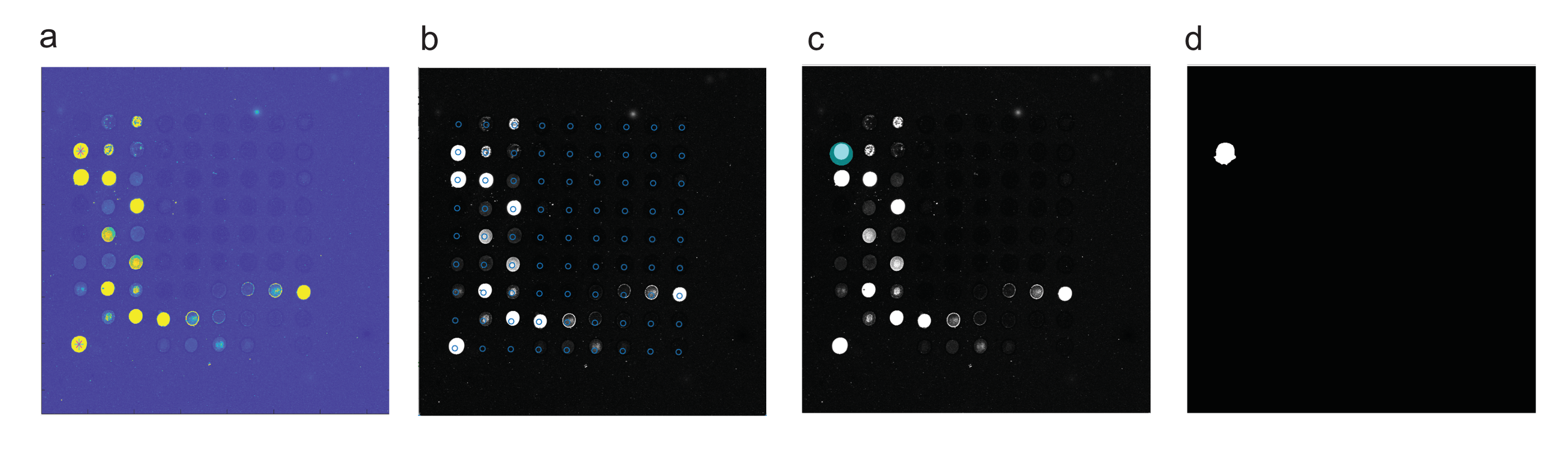

Supplement: S7 Fig — A MATLAB-based algorithm was developed to perform the analysis of the gray scale intensity of the microarray. The original image of the array was uploaded and cut into subarrays. The location of two reference spots was manually adjusted (Fig panel A) and a template of a 9x9 array was placed on top of the array (panel B) and visually confirmed to correspond to the location of the spots. A mask was located on each spot of the array (panel C) and using Otsu thresholding the spot was adjusted (panel D). The gray intensity of each spot was then calculated. Spots presenting artifacts were removed from the analysis. (TIF) [file pone.0259332.s007.tif]

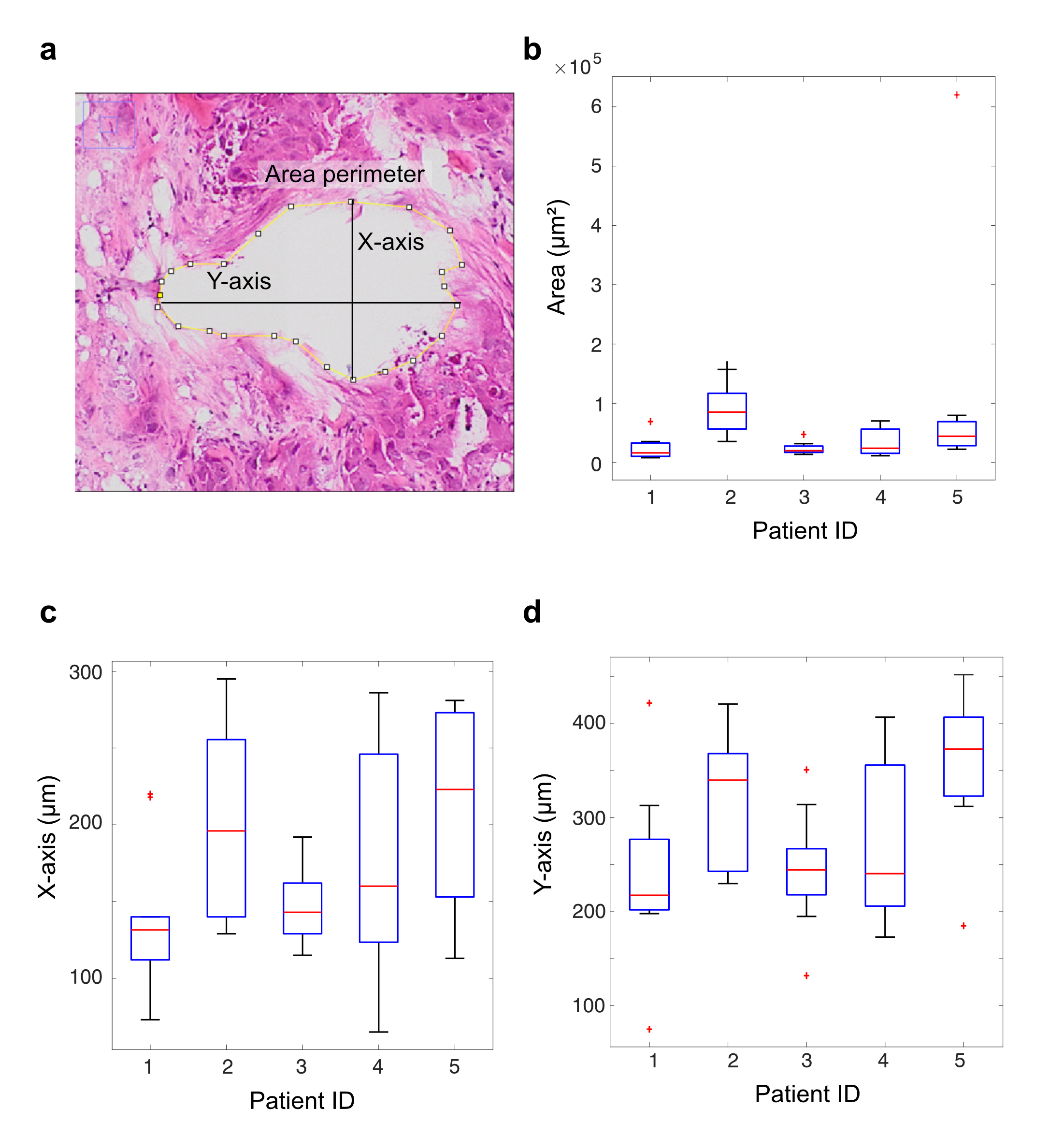

Supplement: S8 Fig — a) Definition of area of the footprint, the X and the Y-axis for analysis. Extracellular matrix was excluded from the area considerations. b, c and d) Boxplot representing the area, X-axis and Y-axis of the footprints for each patient, respectively. Red crosses represent the outliers. (TIF) [file pone.0259332.s008.tif]
