# Supplementary material for: Spatial protein heterogeneity analysis in frozen tissues to evaluate tumor heterogeneity
Source: PLoS One. 2021 Nov 19;16(11):e0259332. doi: 10.1371/journal.pone.0259332 (PMC8604290; doi:10.1371/journal.pone.0259332)
Supplement: S1 Table — (PDF) [file pone.0259332.s009.pdf]

Table S1 | Disease staging of the patients suffering from invasive-ductal breast cancer (IDC).

Patient ID	Analyzed region	Disease staging	ER expression	PR expression
P1	DCIS	pT1c, pN3, G3	100%	30%
P2	IDC	pT2, pN0, G3	>90%	>90%
P3	DCIS and IDC	pT2, pN1a, G3	50%	50%
P4	IDC	pT4b, pN2a, G3	0	0
P5	IDC	No information available	0	0
